# Unlocking autism’s complexity: the *Mov*e Initiative’s path to comprehensive motor function analysis

**DOI:** 10.3389/fnint.2024.1496165

**Published:** 2025-01-21

**Authors:** Ashley Priscilla Good, Elizabeth Horn

**Affiliations:** 2m Foundation, Hillsborough, CA, United States

**Keywords:** autism, wearable technology, sensor-based data, cross-disciplinary research, motor function, real-world data (RWD), autism motor signature (AMS), precision measurement

## Abstract

The long-standing practice of using manualized inventories and observational assessments to diagnose and track motor function in autism overlooks critical data invisible to the naked eye. This subjective approach can introduce biases and hinder the translation of research into clinical applications that rely on objective markers of brain–body connections. Meanwhile, we are experiencing a digital healthcare revolution, marked by innovations in the collection and analysis of electronic health records, personal genomes, and diverse physiological measurements. Advanced technologies, including current wearable devices, integrate both active and passive (sensor-based) data collection, providing a more comprehensive view of human health. Despite advances in sensors, wearables, algorithms, machine learning, and agentic AI, autism research remains siloed, with many tools inaccessible to affected families and care teams. There is a pressing need to merge these technological advances and expedite their translation into accessible, scalable tools and solutions to diversify scientific understanding. In response, this Perspective introduces the *Move* Initiative, a coalition spearheaded by the nonprofit 2 m Foundation, composed of self-advocates, families, clinicians, researchers, entrepreneurs, and investors who aim to advance and refine the measurement of movement in autism. *Move* will make motor screenings more dynamic and longitudinal while supporting continuous assessment of targeted interventions. By fostering cross-disciplinary collaboration, *Move* seeks to accelerate the integration of the expanding knowledge base into widespread practice. Deep, longitudinal, multi-modal profiling of individuals with Autism Spectrum Disorder offers an opportunity to address gaps in current data and methods, enabling new avenues of inquiry and a more comprehensive understanding of this complex, heterogeneous condition.

## 1 The role of motor symptoms in enhancing autism diagnosis and care

### 1.1 Rising autism prevalence and unmet needs

Autism prevalence has risen significantly over the past two decades, with the CDC reporting that 1 in 36 eight-year-olds were diagnosed with Autism Spectrum Disorder (ASD) in 2020 (Maenner et al., 2023). Despite this growing prevalence, there are no FDA-approved treatments for autism or the motor challenges frequently associated with the condition (McGuinness and Kim, 2020). While improved awareness and screening have contributed to the increase in diagnoses, the steady rise in birth-year prevalence—beginning in the late 1980s and continuing into the early 1990s—suggests that additional genetic, environmental, and developmental factors may also be at play (Chang et al., 2023; Escher et al., 2022; Guo et al., 2022; Hicks et al., 2017; Kuja-Halkola et al., 2019; Li et al., 2022; Naviaux, 2020; O’Sharkey et al., 2024; Shenouda et al., 2022; Zahorodny et al., 2023). These trends underscore the need for continued research to unravel the complex mechanisms contributing to ASD.

The rising prevalence of autism also comes with profound societal implications. The economic impact of autism is equally staggering. Costs associated with the condition are expected to soar to $7 trillion by the year 2029, driven by the growing demand for therapies, education, and support services (Cakir et al., 2020). This financial burden, coupled with the lack of effective treatments, highlights the urgent need to adopt innovative approaches that address the overlooked components of autism, such as motor function.

### 1.2 Behavioral diagnosis and the role of the DSM-V

For the past 80 years, autism has been diagnosed based on behavioral criteria (Jackman and Zwaigenbaum, 2023; Qin et al., 2024). In the 1970s and early 1980s, autism was considered rare, with an estimated prevalence of 1 in 10,000 children (Jackman and Zwaigenbaum, 2023). During this period, the condition was often misclassified as other disorders, such as Childhood Schizophrenia, Childhood Affective Disorder, or Pervasive Developmental Delay (Genovese and Ellerbeck, 2022; Jackman and Zwaigenbaum, 2023). The subsequent evolution of diagnostic criteria—culminating in the broader classification of ASD in the DSM-V—reflected advances in understanding and recognition of autism’s heterogeneity (Jackman and Zwaigenbaum, 2023).

Despite advances in understanding autism, diagnosis still relies on observable behavioral symptoms, such as difficulties with social interaction, communication, and repetitive behaviors (American Psychiatric Association, 2013). This behavioral focus often overlooks the physiological underpinnings of autism, including motor and sensory differences, which are increasingly recognized as integral to its presentation (Torres et al., 2016; Licari et al., 2020). The continued reliance on subjective assessments contributes to delayed diagnoses and an incomplete understanding of autism’s complexity. With behaviors ranging widely—from severe physical and mental challenges to relatively mild traits that present as social quirks (Qin et al., 2024; Zwaigenbaum and Penner, 2018)—the underlying causes and most effective treatments remain elusive.

It is important to clarify that we do not advocate for abandoning the DSM-V. Instead, we propose enhancing its utility by incorporating objective, motor-focused data into the diagnostic framework. Motor symptoms, often observed alongside core behavioral characteristics, represent a quantifiable and overlooked domain in autism care. This integration could improve precision and consistency, while preserving the value of behavioral assessments. By adopting a more comprehensive, multi-dimensional approach that includes motor function and physiological data, the field can move beyond the limitations of purely behavioral diagnosis.

### 1.3 Motor function as a foundation for understanding autism

Motor challenges in ASD extend beyond physical impairments, influencing speech, sensory processing, and learning. Since speech itself is a motor activity, difficulties in motor planning and coordination likely contribute to language delays and communication challenges (Brignell et al., 2018). Research underscores the connection between sensory perception, motor skills, and cognitive processes, with many individuals with autism exhibiting deficits in fine and gross motor functions (Bhat et al., 2011; Kangarani-Farahani et al., 2024; Lim et al., 2021; Zampella et al., 2021). Sensorimotor integration difficulties—disruptions in perceiving, processing, and responding to sensory information through motor actions—are linked to sensory sensitivities, repetitive behaviors, and communication deficits (Torres et al., 2013; Whyatt, 2017; Donnellan et al., 2013). Variability in movement, including repetitive motions and motor planning difficulties, further compounds these challenges, affecting both verbal and nonverbal communication. (Torres and Whyatt, 2017) Many individuals with ASD remain minimally verbal, with 25 to 30% of children failing to develop functional language, highlighting the motor basis of speech impairments (Brignell et al., 2018; Torres and Donnellan, 2015; Whyatt and Craig, 2013).

With autism prevalence likely exceeding 2.78% of the population (Maenner et al., 2023), understanding and addressing the motor-related underpinnings of this condition is critically important. Given the strong link between motor function and other core symptoms of ASD, there is an urgent need for practical solutions that can comprehensively capture and analyze motor data to inform both diagnosis and treatment. Collecting and analyzing longitudinal movement and micro-movement data could shed light on precisely how motor function influences the cognitive performance and social behavior of individuals with ASD.

This quantified view of motor function would allow researchers to move beyond subjective behavioral assessments, offering an objective connection between motor skills and the broader range of symptoms currently classified as “autistic behaviors” (American Psychiatric Association, 2013; Genovese and Ellerbeck, 2022; Sellick et al., 2021; Wang et al., 2021). By addressing motor function as a foundational component of autism research and care, the field has the potential to improve diagnosis, enable earlier intervention, and develop targeted therapies that address an underexamined aspect of autism.

### 1.4 The complexity of autism comorbidities

Research consistently shows that ASD is frequently accompanied by a wide range of serious comorbidities, many of which significantly impact the daily lives of affected individuals. The literature supports the assertion that autism is frequently accompanied by serious comorbidities such as seizures, gastrointestinal issues, and allergies (Al-Beltagi, 2021; Burns et al., 2023; Kim et al., 2023; Saleh and Adel, 2019). More broadly, motor difficulties—including challenges with coordination, gait, and balance—are among the most common issues occurring alongside ASD (Matson et al., 2011; Papadopoulos et al., 2014; Vogindroukas et al., 2022). According to parent-reported data from the Simons Foundation, a leading nonprofit in the autism field, approximately 87% of children aged 5 to 15 years with ASD experience difficulties with gross and fine motor control, coordination, postural stability, and balance (Bhat, 2020). Despite these findings, much of autism research has focused on genetics and behavior, largely overlooking one of ASD’s most impactful symptoms—motor difficulties—that significantly autism quality of life.

As highlighted in a 2020 Spectrum article: “We describe what experts know about the causes, characteristics, and consequences of motor difficulties, which they say are ‘among the least understood and most neglected aspects of autism’” (Schenkman, 2020). Researchers studying motor function in ASD have long recognized that movement differences are both pervasive and fundamental to the diagnosis (Alsaedi, 2020; Bougeard et al., 2021; Licari et al., 2020; Melo et al., 2020; Nordin et al., 2021; Posar and Visconti, 2022). Notably, motor abnormalities are among the most common physical comorbidities in autism, with a prevalence as high as 79%, and motor impairments reported to range between 50 and 85% (Christensen and Zubler, 2020; Bhat, 2020).

### 1.5 The need for objective, longitudinal motor data

The long-standing reliance on manualized inventories and subjective observational assessments to evaluate motor function in autism overlooks subtle neuromotor differences that are critical for understanding the condition’s complexity. As Torres et al. (2023) highlights, these traditional methods often fail to capture the nuanced physiological underpinnings of autism, such as micro-movements and sensorimotor variability, which can provide valuable insights into brain–body connections. This subjective approach introduces biases and limits the ability to develop effective, individualized interventions.

Emerging tools such as wearable devices, motion sensors, and longitudinal data collection systems offer a pathway to address these limitations (Torres et al., 2023). By integrating objective motor data into diagnostic and care models, researchers and clinicians can better identify autism subtypes, tailor interventions, and track their efficacy over time. Shifting to precision-based approaches rooted in quantifiable data not only addresses the fragmented nature of autism research but also facilitates interdisciplinary collaboration, advancing our understanding of the complex, heterogeneous nature of ASD.

### 1.6 The fragmented nature of autism research

Historically, autism research has been siloed, with experts in genetics, behavior, environment, and physiology often working in isolation. This fragmentation limits the ability to create a cohesive, holistic understanding of autism’s complex presentation. While these focused efforts have led to important discoveries, this siloed approach has limited the ability to develop comprehensive solutions for the diverse challenges of ASD. The multifaceted nature of autism—spanning motor, sensory, cognitive, and environmental domains—demands a more integrated, collaborative approach across fields.

Pellicano and Stears (2011) argue that addressing these unmet research needs requires interdisciplinary collaboration, synthesizing knowledge and methods from diverse fields to tackle the complexity of autism. Similarly, Lord et al. (2022) emphasize the importance of multi-dimensional data and integrated care models to advance understanding and treatment. By combining insights from motor science, technology, and neurobiology, researchers can develop more holistic frameworks that recognize the interconnected nature of ASD symptoms. Addressing these silos is essential to uncovering new avenues for precision diagnosis and intervention.

## 2 Leveraging technology for precision autism care

### 2.1 Technological advancements in health tracking

The last decade has seen significant advancements in digital tools capable of collecting high-resolution, longitudinal health data. Devices such as wearables, environmental sensors, and health-tracking applications have fundamentally changed how we gather and analyze information about human activity. Gary Wolf, co-founder of the Quantified Self movement, introduces the concept of *Personal Science*, which emphasizes the use of empirical methods and real-world data to investigate personal health questions while uncovering patterns in behavior and well-being (Wolf and De Groot, 2020). These advancements have shifted self-tracking from anecdotal observations to systematic, data-driven insights, enabling individuals to uncover subtle quantitative patterns in their behavior and health.

For individuals with ASD, these same technological tools offer an unprecedented opportunity to monitor motor function. Devices such as smartwatches, accelerometers, and EEG wearables can capture nuanced motor behaviors and sensory-motor variability over time. This longitudinal data is essential for identifying motor-based subtypes of autism, understanding developmental trajectories, and personalizing care for individuals with diverse needs.

### 2.2 The case for precision autism care

Traditional, one-size-fits-all approaches to autism care have proven insufficient to address its variability (Lord, 2019; Torres, 2021). Instead, a precision-based model of care—which integrates real-time motor data and other objective metrics—offers the potential to provide personalized baselines for diagnosis, monitoring, and intervention. Dr. Catherine Lord of UCLA underscores this need for precision data, noting that while numerous interventions exist for autism, there is insufficient information about “which treatments or services should be offered, to whom, when, for how long, with what expected outcomes, and for what cost” (Lord et al., 2022). Digital health technologies are well-positioned to close this gap, allowing researchers and clinicians to integrate multi-dimensional datasets—including motor patterns, biometrics, and environmental exposures—into actionable care pathways.

### 2.3 Digital systems: capturing longitudinal motor data

**Figure 1:**
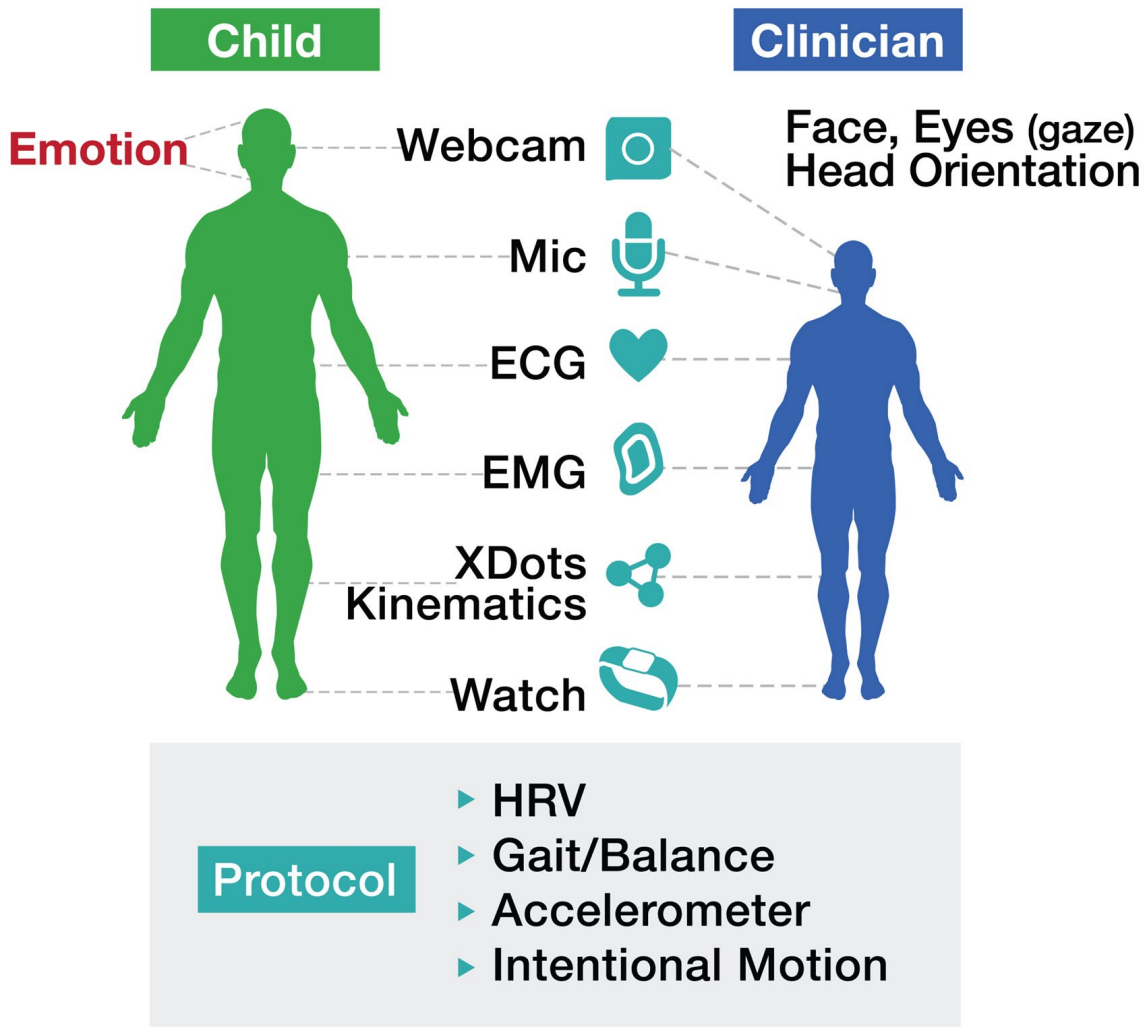
Motor protocol illustrates a comprehensive, technology-driven approach to assessing motor function in individuals with autism. The protocol incorporates tools such as wearables, webcams, ECG, and accelerometers to track metrics including heart rate variability (HRV), gait/balance, and intentional motion. Importantly, this approach emphasizes the collaborative interaction between the child and clinician, leveraging real-time data to inform interventions and enhance outcomes.

The integration of “long and wide data,”—terms introduced by Wolf and De Groot (2020) —into autism care frameworks has the potential to reshape how we identify and track motor challenges. Longitudinal data—collected passively over extended periods through wearables and sensors—enables the detection of subtle changes in movement patterns that might otherwise go unnoticed. Simultaneously, wide data—which incorporates multiple dimensions such as motor function, EEG signals, environmental exposures, and genetic information—provides a holistic view of autism’s complexities. By combining these approaches, digital systems can transition from static, categorical assessments to dynamic, individualized baselines that reflect the evolving needs of individuals with ASD. This shift not only improves the precision of diagnostic and treatment protocols but also highlights the significant role of motor function in influencing broader developmental trajectories and behaviors (see Figure 1).

**Figure 2:**
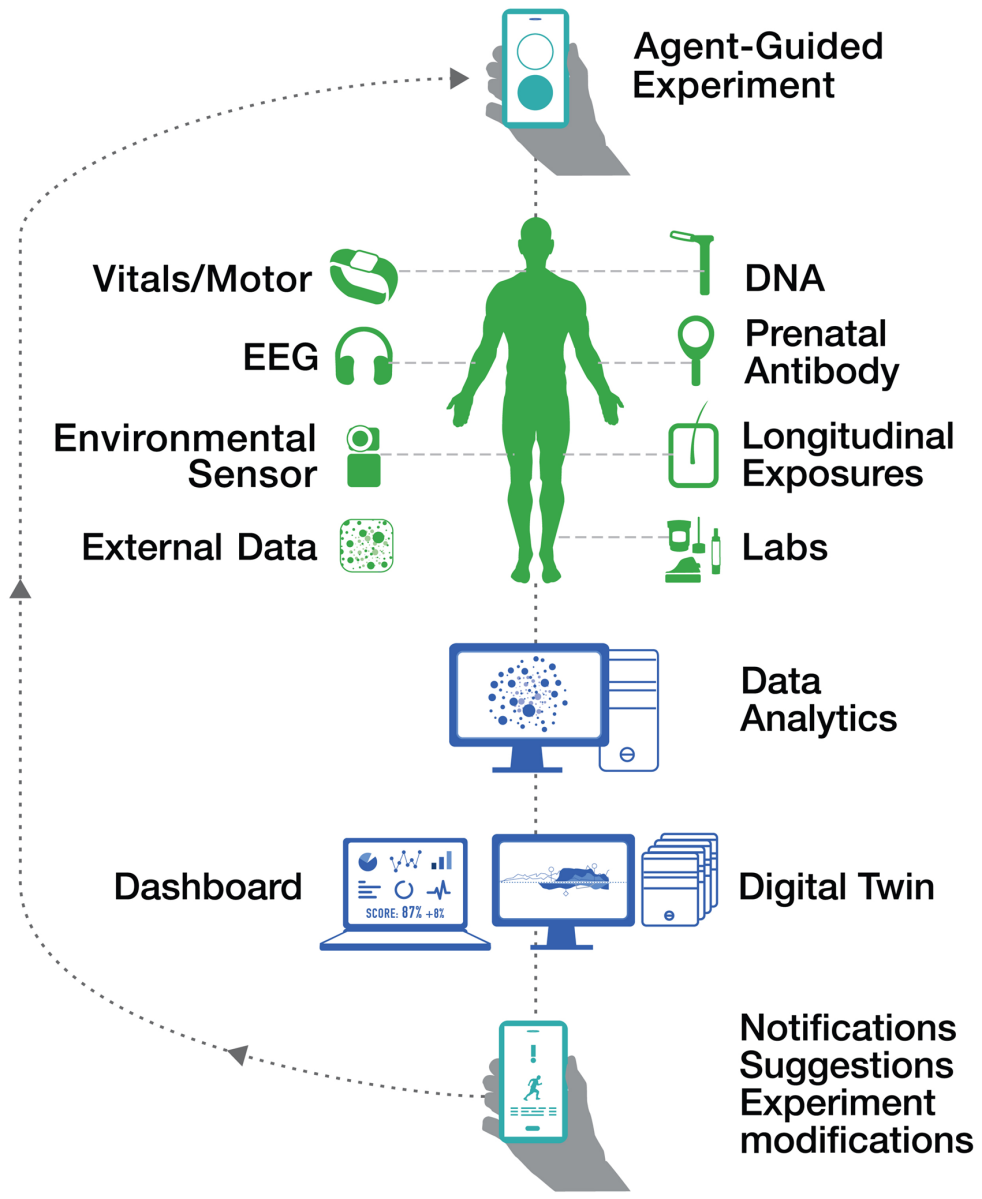
Decoding autism cycle.

This iterative process is visually represented in Figure 2 Decoding Autism Cycle. The figure highlights how diverse data sources—including vitals, motor activity, EEG, DNA, longitudinal exposures, and external data—are integrated into a digital twin framework. Through personalized dashboards and real-time notifications, actionable insights enable targeted experiment modifications, fostering continuous improvement in motor function analysis and intervention strategies.

While advanced technologies offer immense potential for capturing longitudinal motor data, many tools remain inaccessible to affected families and care teams. Barriers such as cost, limited technical knowledge, and the absence of autism-specific design prevent widespread adoption of wearable devices and health-tracking platforms. For example, a meta-analysis by Xu et al. (2024) highlights that while digital interventions can support developmental skills in individuals with ASD, accessibility challenges and usability limitations hinder their effective use by families. Similarly, a study by Ning et al. (2019) identified significant limitations in access to autism-related resources, particularly for families in rural areas, highlighting the urgent need for accessible and intuitive digital health solutions. Developing cost-effective systems tailored to the unique needs of individuals with ASD and their families is essential to ensure these technologies can be seamlessly integrated into daily life and clinical care.

### 2.4 From data to action: harnessing machine learning and artificial intelligence

While collecting longitudinal data is an essential first step, its true value lies in analysis and interpretation. Machine learning (ML), artificial intelligence (AI), and natural language processing enable the real-time analysis of vast datasets, uncovering correlations between motor function and other developmental domains, identifying motor-based subtypes of autism, and generating actionable insights for clinical care. For example, ML and AI technologies have been instrumental in identifying motor patterns and predicting disease progression in neurological disorders such as Parkinson’s Disease and Multiple Sclerosis, using tools like wearables and advanced imaging (di Biase et al., 2024; Lonini et al., 2018; Zhan et al., 2018). These existing frameworks demonstrate how AI-driven tools can facilitate the recognition of subtle, individualized motor signatures and inform precision interventions.

Personalized health dashboards further enhance this process by providing user-friendly tools to visualize motor trajectories, monitor progress, and adjust interventions in real time (Snyder and Zhou, 2019). In autism, similar systems could aggregate fine and gross motor data to uncover critical patterns, such as micro-movements or postural shifts, that may correlate with behavioral or cognitive milestones. (Torres and Whyatt, 2017) These systems not only support individual-level care but also contribute to global, open-source datasets, enabling population-level studies that address the heterogeneity of ASD. By building on existing successes in neurological research, the application of ML and AI in ASD has the potential to revolutionize how motor function is understood, monitored, and managed.

### 2.5 Addressing accessibility and design considerations

While wearable technologies offer immense potential, it is essential to recognize the unique needs of individuals with ASD, including those with heightened sensitivity to sensory inputs or electromagnetic fields (EMFs). Research has suggested that individuals with autism may exhibit increased vulnerability to EMF exposures, potentially due to underlying biological susceptibilities, including oxidative stress, neuroinflammation, and mitochondrial dysfunction (Herbert and Sage, 2013a; Herbert and Sage, 2013b). These sensitivities can result in discomfort or exacerbate symptoms, highlighting the need to design wearable devices that mitigate non-native radiofrequency (RF) exposure and accommodate the sensory needs of individuals on the autism spectrum (Herbert and Sage, 2013b; Pall, 2016). This underscores the importance of designing wearable devices that mitigate non-native RF exposure and accommodate the specific sensory needs of individuals on the autism spectrum (Herbert and Sage, 2013b).

To address these concerns, it is critical for manufacturers to prioritize design features that reduce RF emissions, particularly for technologies that rely on constant data transmission. A practical solution is the inclusion of an ‘airplane mode’ option, which allows users to limit exposure to non-native EMFs by disabling wireless connectivity while maintaining local data collection capabilities. Such solutions have been recommended to reduce RF emissions, particularly for sensitive populations, including individuals with ASD (Sage and Burgio, 2018; Pall, 2016). This feature, already standard in many consumer devices, could be adapted to additional wearable technologies to promote accessibility for individuals who experience discomfort or adverse effects related to EMFs. By enabling individuals and care teams to control RF exposure on demand, airplane mode represents a simple yet meaningful modification that aligns with the sensory and biological considerations of the autism community.

In addition to RF considerations, wearable technologies must be designed with attention to physical comfort, ease of use, and adaptability. Features such as lightweight materials, adjustable components, and user-friendly interfaces are essential for accommodating motor and sensory challenges. Tailored approaches to wearable design can significantly improve usability and adoption in individuals with autism, particularly when end-users and their families are involved in the design process (Ning et al., 2019). Collaboration with individuals on the autism spectrum, as well as their families and care teams, is critical in refining these technologies to make them both functional and inclusive.

### 2.6 Toward a new era of autism research

The rise of wearable sensors, digital health platforms, and AI-driven analytics represents a pivotal opportunity for advancing autism care. By capturing longitudinal motor data and integrating it with other biological and behavioral metrics, current technologies allow for a more precise, data-driven understanding of ASD. This approach not only has the potential to improve subtyping and diagnosis but also to personalize interventions in ways that meet the evolving needs of individuals across the spectrum. Through the integration of technology, we can move closer to a future where autism care is informed by real-world, longitudinal evidence, empowering individuals and their care teams with tools for meaningful change.

## 3 The *Move* Initiative: advancing motor function analysis in autism

### 3.1 Introducing the *Move* Initiative

Building on the technological advancements and tools discussed in Section 2, the *Move* Initiative translates these innovations into practical applications that address critical challenges in autism motor research and care. Organized and led by the 2m Foundation, this collaborative project aims to advance the study and analysis of motor function in autism. As a nonprofit focused on patient-led, technology-driven solutions for chronic conditions, 2m Foundation has brought together an interdisciplinary coalition of self-advocates, families, care teams, clinicians, researchers, and technology developers to tackle disparities in motor research. The initiative emphasizes integrating motor data into diagnostic and care models while fostering collaboration across disciplines such as motor science, neurobiology, digital health, and artificial intelligence. By breaking down traditional research silos, *Move* establishes an open, collaborative platform for sharing data and developing practical tools.

Central to the *Move* Initiative is the development of an Autism Motor Signature (AMS)—a framework aimed at capturing individualized motor function profiles over time. While still in progress, the AMS aspires to analyze micro-movement patterns across a cohort of individuals on the autism spectrum, leveraging wearable devices, motion sensors, and digital health platforms to continuously collect fine and gross motor data. Similar to how tremor and dyskinesia signatures in Parkinson’s Disease have been identified using tools like the Apple Watch and Rune Lab’s StrivePD app (Larkin, 2022), the AMS seeks to uncover distinctive motor patterns indicative of ASD. These global analyses, powered by AI-driven tools, could lead to the identification of unique motor-based signatures for ASD, informing personalized interventions and enabling targeted care strategies. By integrating this data, the AMS framework supports actionable care models, allowing individuals and their care teams to refine interventions and monitor outcomes in real time (Mandelli et al., 2024; Iannone and Giansanti, 2023).

### 3.2 A data-driven approach to personalized care

Motor function exists within a broader biological and environmental mosaic, shaping autism’s developmental pathways. As Topol (2015) explains, “You have to be able to connect the dots, the whole mosaic.” This multi-omic approach integrates motor data with other variables such as sleep, diet, genomics, and environmental exposures, creating a holistic framework for understanding ASD.

The *Move* Initiative aims to leverage these interconnected data streams to generate actionable insights for precision autism care. By moving beyond one-size-fits-all therapeutic frameworks, this approach enables individuals to quantify motor symptoms, test interventions, and track progress. Personalized health dashboards provide user-friendly tools for visualizing motor trajectories, empowering caregivers and clinicians to make informed decisions in real time (Snyder and Zhou, 2019). Aggregated data further contributes to global, open-source datasets, advancing population-level studies and addressing the heterogeneity of ASD.

### 3.3 Future directions for motor function analysis

While the AMS is still in development, its potential to transform precision autism care is vast. Aggregated motor data, analyzed alongside biological and environmental factors, will contribute to identifying ASD subtypes and developing targeted interventions (Ferrari, 2021; Joudar et al., 2023). AI-driven tools and intuitive dashboards will ensure that motor data is accessible to families, clinicians, and researchers, facilitating real-world applications. Through its emphasis on collaboration, real-world data, and scalable solutions, the *Move* Initiative represents a significant step forward in addressing the motor challenges of autism. By advancing our understanding of how motor function shapes the developmental landscape of ASD, this effort lays the groundwork for more comprehensive and personalized care for individuals with autism.

As the *Move* Initiative progresses, its success depends on the collective expertise and shared insights of a multidisciplinary community. Researchers, clinicians, engineers, and advocates are invited to join this effort, contributing their knowledge to refine tools, expand datasets, and develop actionable solutions. By working together, we can accelerate progress toward a future where motor function analysis is an integral component of autism care, ultimately improving outcomes for individuals with ASD and their families.

## 4 Concluding remarks

The *Move* Initiative, coordinated by the 2m Foundation, exemplifies a transformative step in addressing the motor challenges of Autism Spectrum Disorder (ASD). By integrating tools such as wearable devices, sensor-based data collection, and AI-driven analytics, the initiative develops dynamic, longitudinal models of care tailored to the diverse needs of individuals with autism. Central to this effort is the Autism Motor Signature (AMS), a precision framework designed to quantify motor function and uncover ASD subtypes, fostering interventions that are both personalized and impactful.

This perspective article provides a framework for understanding how motor function analysis can be integrated into diagnostic and care models to advance both clinical and scientific understanding of ASD. By prioritizing accessibility and scalability, the *Move* Initiative unites self-advocates, families, clinicians, researchers, and technologists to break traditional research silos and translate insights into actionable, real-world solutions. Through innovation and collaboration, this work paves the way for a future of tailored, evidence-based support for individuals with ASD.

## Data Availability

The original contributions presented in the study are included in the article/supplementary material, further inquiries can be directed to the corresponding author.
